# Association between chronic stress and the epigenome: Exploration of psychological and biological stress

**DOI:** 10.1371/journal.pone.0346517

**Published:** 2026-04-06

**Authors:** Melissa Miller, Robin D. Brown, Cole Krautkramer, Rhea Gogia, Hagen Fritz, Dennis Wylie, Kerry Kinney, Frances A. Champagne

**Affiliations:** 1 Department of Psychology, University of Texas at Austin, Austin, Texas, United States of America; 2 Department of Psychiatry, University of North Carolina, Chapel Hill, North Carolina, United States of America; 3 Department of Civil, Architectural and Environmental Engineering, University of Texas at Austin, Austin, Texas, United States of America; 4 Center for Biomedical Research Support, University of Texas at Austin, Austin, Texas, United States of America; Chuo University, JAPAN

## Abstract

Chronic stress is a predictor of health outcomes. Epigenetic mechanisms such as DNA methylation (DNAm) have been proposed as mediators between stress and health trajectories, with evidence from animal models supporting a causal relationship between chronic stress and variation in the epigenome. However, when examining the impact of stress in humans, stress is often very broadly defined and includes both the perception of stress (psychological stress) and biological indices of stress such as variation in the stress hormone cortisol (biological stress). There has been limited exploration of the distinct epigenetic associations with psychological vs. biological stress or analysis of these relationships that consider genome-wide DNAm. The current study used a longitudinal design (9 weeks) with 69 healthy participants aged 18–35. Self-reported psychological stress was assessed several times a week and averaged over the 9-week period. Hair cortisol concentration (HCC) and salivary genome-wide DNAm were assessed at the end of the 9-week period. We identified epigenomic associations between chronic psychological stress (956 CpG sites) and HCC (10,335 CpG sites), with elevated chronic psychological stress generally associated with DNA hypomethylation and elevated HCC generally associated with DNA hypermethylation. Self-report stress and HCC were not correlated. DNAm at 16 CpG sites was significantly associated with both psychological stress and HCC, though in opposing directions which may suggest potential upregulation vs. downregulation of overlapping biological pathways identified in response to these stressors. Associations between stress (psychological and biological) and DNAm within stress-associated genes were observed, generally suggesting a positive association between stress and DNAm. Epigenetic age (PhenoAge) was negatively associated with psychological stress and HCC, though these relationships were sex-specific and varied based on ethnicity-race. Findings suggest unique associations between stress and the epigenome, highlighting the complex pathways through which the biological embedding of stress and subsequent stress-associated health outcomes may emerge.

## Introduction

Chronic stress experienced in early life and in adulthood has been associated with an increased risk of psychopathology and negative health outcomes [1–4]. Investigation of the pathways through which chronic stress exerts these long-term effects has implicated the activation of hypothalamic-pituitary-adrenal (HPA) axis, which increases allostatic load indicating increased “wear and tear” on physiological systems [5]. While HPA activity serves an adaptive function by mobilizing energy and metabolism to respond to stressors, chronic HPA activation that persists beyond the timing of stress exposure may lead to neuroendocrine and immune dysregulation. The lasting impact of chronic stress may be a consequence of stress-induced molecular changes that alter gene expression and cellular functioning such as DNA methylation (DNAm). The covalent attachment of a methyl group at a CpG dinucleotide is the most commonly studied type of epigenetic modification and has been associated with psychopathology [6–9]. Studies in animal models demonstrate that chronic stress exposure induces changes in gene expression and DNAm of stress-related genes [10,11]. Thus, it has been proposed that epigenetic modifications such as DNAm mediate the link between stress exposure and health outcomes [5,12]. Evidence of an association between stress and variation in the epigenome has also been observed in humans [13,14]. However, the existing research is limited by broad definitions of psychological and biological experiences of stress.

The psychological experience of stress is often assessed through self-report measures that assess the feelings of being stressed, nervous, or irritable, being unable to cope with life events, and being unable to control life events and emotional reactions to those events [15]. Psychosocial stress is a predictor of negative health outcomes, including cardiovascular disease [16], obesity [17], and depression and anxiety [18]. Among individuals who are exposed to chronic stress, the perception of being stressed is a significant predictor of immune functioning [19]. Exposure to persistent psychological stressors, such as low socioeconomic status or work burden, are associated with altered DNAm and acceleration of epigenetic aging [20–22]. Elevated perceived psychological stress has been found associated with decreased DNAm of the methylenetetrahydrofolate reductase (*MTHFR*) gene, which is also associated with depression [23], and the relationship between perceived psychological stress and suicidality has been found to be mediated by altered DNAm [24]. These studies suggest that the perception of stress plays a critical role in the association between stress, DNAm, and health outcomes. However, the distinct associations between perception of stress and the epigenome have yet to be explored within a study design that also considers the impact of stress on HPA activation.

In humans, exposure to stress is associated with increases in cortisol levels that have typically been measured within blood or saliva. While cortisol is commonly thought of as an indicator of HPA activity, circadian variation impacts plasma and saliva derived cortisol levels [25]. Thus, cortisol levels obtained from plasma and saliva samples may not serve as a strong predictor of *chronic* HPA output. However, there is increasing use of hair cortisol concentration (HCC) as a measure of cortisol that may be more appropriate for studies assessing chronic biological stress [26,27]. Elevations in HCC have been observed in association with chronic stress occurring during childhood [28,29] and adulthood [27,29]. Mental health outcomes are associated with HCC, though the direction of this relationship varies dependent on the specific diagnosis, with depression associated with increased HCC and anxiety associated with decreased HCC [30]. The potential mediating role of DNAm in the link between HCC and health outcomes has been proposed, though investigation of these relationships has typically focused on candidate genes. For example, in adult participants, elevated HCC has been associated with decreased DNAm within the serotonin transporter gene (*SLC6A4*) [31], whereas elevated HCC has been associated with increased DNAm within the brain derived neurotrophic factor gene (*BDNF*) [32]. While there has been some exploration of HCC and genome-wide DNAm during infancy [33], less is known about the relationship between chronic biological stress and genome-wide DNAm in adulthood. Further, evidence indicating a lack of association between HCC and the psychological perception of stress [34] highlight the importance of considering the convergent and divergent epigenomic associations with psychological and biological stress.

In the current study, we investigate the association between chronic psychological stress (self-report perception of stress), chronic biological stress (HCC), and genome-wide DNAm in saliva samples from healthy young adults participating in a 9-week assessment period. Our analyses explore epigenome-wide associations, relationships between stress and DNAm in stress-related candidate genes, and epigenetic age. To the best of our knowledge, this is the first study to investigate these relationships using longitudinal data on a genome-wide scale. We predicted that DNAm would be significantly associated with both chronic psychological stress and biological stress, with unique epigenomic associations emerging with each stress measure.

## Materials and methods

### Participants

Participants were students at the University of Texas at Austin and were required to be 18–35 years of age and have at least 3 cm of hair at the posterior vertex. Participants were prescreened and excluded if taking endocrine-related medication (with the exception of oral contraceptives), having current substance abuse, or current use of nicotine products. To reduce the potential impact of broad reproductive hormone shifts, females were excluded if pregnant, breastfeeding, or if they had an irregular menstrual cycle. Participants who completed all components of the study received course credits, a Fitbit®, or were entered in a drawing for $20 USD.

### Ethics statement

This study was approved by the Institutional Review Board at the University of Texas at Austin (Protocol# 2019090120) and conformed to the guidelines and regulations outlined by this Institutional Review Board. Recruitment of subjects in this study commenced following IRB approval in November 2019 with data collection starting in January 2020 and ending in March 2020. All participants were over 18 years of age and gave written consent to participate in the study. Consent forms were signed during an in-person visit to the lab and stored in a secure location. Participants were provided with information about the study, and the consent process was witnessed by research staff.

### Study protocol

The study protocol involved participant enrollment into a 9-week assessment period, during which indices of psychological and biological stress were measured, with a salivary DNA sample collected at the study endpoint. At the baseline visit (January 2020), participants were instructed through the setup of Beiwe [35], an open-source mobile phone application that was used to administer ecological momentary assessment surveys. Participants were instructed to answer the psychological stress surveys as soon as they received a notification and the mobile data obtained from this application was linked to the specific participant via a hashed identifier incorporated into the application [35]. After approximately 9 weeks (March 2020), participants provided a hair sample for cortisol assay and a saliva sample for DNA extraction. REDCap [36], a secure data capture application, was used to collect demographic data.

### Psychological stress assessment

#### Ecological Momentary Assessment (EMA).

Ecological momentary assessment (EMA) was used to collect real-time self-reported psychological stress data throughout the study period (January 2020 to March 2020). Participants were prompted with a brief EMA survey asking them to answer a four-point Likert scale question about their momentary psychological stress level. To minimize respondent burden, the EMA survey was only administered in the morning (7 a.m.) and evening (7 p.m.) on Monday, Wednesday, Friday, and Sunday of each week. Participants were prompted with “RIGHT NOW, I am feeling STRESSED” and given the following responses to choose from: “Not at all”, “A little bit”, “Quite a bit”, and “Very much”. EMA stress responses were encoded to integer values between 0 (no stress) and 3 (high stress), respectively. Given the highly significant Pearson correlation between the weekly average morning and average evening EMA stress data (r = 0.78, t = 57.991, df = 2192, p < 2.2 e-16, 95% CI: 0.761–0.794), morning and evening daily EMA stress data was combined for each participant. To increase statistical power, EMA stress was then averaged across the entire study period for each participant to generate an index of self-reported psychological stress.

#### Perceived Stress Scale (PSS).

The 10-item Perceived Stress Scale (PSS) [15], which retrospectively measures self-reported perceived stress in the last month, was utilized as a secondary measure of psychological stress to determine if EMA responses correspond with this validated measure of stress. The PSS was administered weekly on Saturdays mornings (7 a.m.). Weekly PSS scores were averaged for the entire study period for each participant.

### Biological stress assessment using hair cortisol concentration (HCC)

#### Hair collection.

After the 9-week assessment period, hair samples were collected for HCC analysis. Information on participants current hair care and treatment routine was collected [34]. Hair length was assessed and approximately 150–200 strands of hair of at least 3 cm in length were collected from participants by cutting hair samples at the scalp of the posterior vertex using cleaned barber shears. The hair was then placed in aluminum foil and the scalp end of the hair was indicated with an arrow marked on the foil. For participants with long hair, a hair sample from one area of the posterior vertex was collected. For participants with shorter hair, 2–4 small clusters of hair were collected from different locations at the posterior vertex to maintain the appearance of the hair after collection. Hair samples were stored at room temperature until further processing.

#### Processing hair samples.

The first 3 cm of hair closest to the scalp were processed for all samples according to recommendations outlined in detail elsewhere [37]. Briefly, hair samples were washed in 5 mL of HPLC-grade isopropanol while rotating for 3 minutes at room temperature, then centrifuged at 1500 x g for 1 minute. The isopropanol was then discarded, and the hair wash step was repeated for a total of 2 washes. Hair samples were air dried by placing open sample tubes in a fume hood for at least 48 hours. Up to 60 mg of hair was then added to its corresponding reinforced microvial along with three 0.2 mm chrome steel beads. The hair was then ground for 3 minutes using a Mini-BeadBeater-16 (BioSpec Products). Sample tubes were visually inspected to ensure that the hair was thoroughly ground. 1.5 mL of HPLC-grade methanol was then added to the hair samples with constant inversion on a rotator at room temperature for 24–36 hours. Samples were then centrifuged at 10,000 rpm at room temperature for 5 minutes to allow for separation of the supernatant. 1 mL of the supernatant was transferred to a 1.5 mL microcentrifuge tube and concentrated in a SpeedVac™ DNA130 Vacuum Concentrator (Thermo Fisher Scientific) for 45–60 min then stored at −20°C. Dried samples were reconstituted in 200–400 mL of assay buffer and HCC was measured in duplicate using a competitive enzyme-linked immunosorbent assay (ELISA) as specified by the manufacturer’s instructions (Enzo Life Sciences). The Biotek Synergy LX micro-plate reader (Agilent Technologies) was used to measure the optical density at 405 nm with a correction at 580 nm. The concentration of cortisol was calculated using a 4-parameter logistic curve using the plate reader’s Gen5 software. The sensitivity of the assay was 56.72 pg/mL, the intra-assay coefficient of variation was 7.3–10.5%, and the inter-assay coefficient of variation was 8.6–13.4%. The assay specificity for cortisol was 100%, the assay cross reactivity for progesterone was 3.64% and <1% for testosterone and estradiol.

#### Hair cortisol analysis.

HCC values were removed if the coefficient of variation (CV) was greater than 20% (based on the total samples processed) or if the sample was outside of the standard curve (n = 13 samples). The mean concentration from the Gen5 software was converted from pg/mL to µg/dl then corrected for mg of hair, mL of methanol, mL of supernatant, and mL of assay buffer, resulting in the final HCC concentration per unit weight of powdered hair of pg/mg. Given that the raw HCC data were positively- (right-) skewed and thus not normally distributed, HCC values were natural log-transformed to normalize the distribution [38]. Statistical analyses were conducted on the transformed HCC data and are thus reported without units [39], however, means and standard deviations are reported in non-transformed units (pg/mg).

### Genome-wide DNA methylation (DNAm)

#### Saliva collection.

After the 9-week assessment period, saliva samples were collected to measure DNAm using the Oragene-500 collection kit per the manufacturer’s instructions (DNA Genotek). Saliva collection was monitored by a researcher to preserve the integrity of the sample. After collection, saliva samples were immediately transferred to Corning™ cryogenic vials and stored at −80°C until DNA extraction.

#### DNA extraction.

Prior to extraction, samples were thawed on ice then centrifuged at 1800 × g at 4 °C for 5 min. 1 mL of saliva was extracted using the MagMAX™ DNA Multi-Sample Ultra 2.0 Kit (Thermo Fisher Scientific) by following the large volume high-throughput automated DNA purification workflow on the KingFisher™ Flex System. Extracted DNA was eluted in 150 µL of elution solution and stored at −20°C.

#### Genome-wide DNA methylation assay.

Salivary DNA methylation levels were determined using the Infinium MethylationEPIC v1.0 BeadChip kit (Illumina) at the Genomic Sequencing and Analysis Facility (GSAF) at the University of Texas at Austin. Samples with at least 500 ng of DNA first underwent sodium bisulphite conversion using the EZ-96 DNA Methylation™ Kit (Zymo Research) according to the manufacturer’s protocol. Bisulfite-converted DNA samples were then loaded onto each MethylationEPIC BeadChip slide. Samples were then processed per Illumina’s Infinium HD Methylation Assay protocol, which measures DNA methylation at over 850,000 CpG sites [40,41]. BeadChips were then scanned using the Illumina NextSeq 550 scanner which measures the fluorescence intensities and stores the data as IDAT files.

#### Preprocessing of DNA methylation data.

Processing of the EPIC BeadArray IDAT image files and data analysis was performed using RStudio; primarily with the minfi package [42]. The data was first examined for low signal intensity then Illumina control metrics were evaluated using the ewastools package [43]. Predicted sex from DNA methylation of the sex chromosomes was verified against survey demographic data. Background correction with dye-bias normalization was carried out using preprocessNoob [44]. Beta (β) methylation values, which is the estimated proportion of cells in a sample that are methylated at a given CpG site, was then determined from the fluorescent signal intensity and used for subsequent analyses. Q-Q plots were used to identify p value inflation (see S1 Fig). Principal component analysis (PCA) was used to identify batch effects by linear regression analysis of the principal components and beta values as well as visual inspection of the PCA plots (see S2 Fig).

Detection p-values, used to assess background fluorescence, were calculated and 1.4% of probes were found to be above a threshold of 0.01. In addition, the number of hybridizing beads measuring DNAm at each CpG site (nbeads) were calculated and 0.16% of probes were found to have low bead numbers (n < 4). 44,063 CpGs, for which 5% of samples were flagged for detection p-values or nbeads, were removed. All samples had fewer than 10% unreliable probes and thus no samples were filtered. 26,731 probes with a SNP in the CpG were also removed. 39,724 cross-reactive probes were excluded, and 4,220 gap probes were removed.

Saliva consists of epithelial cells and immune cells present in the oral cavity [45], with each cell type contributing its own unique DNAm signature. Saliva cell proportions were estimated using the Bioconductor BeadSorted.Saliva.EPIC package developed from a reference panel of Illumina EPIC data from BeadSorted saliva cells [46], which was implemented using the ewastools package function estimateLC (https://github.com/hhhh5/ewastools). We have previously found this method to have high concordance with cell type proportions estimated using the EpiDISH deconvolution algorithm [14].

Downstream analysis was focused on autosomal chromosomes and thus 11,794 sex probes were further excluded from the analysis. Sentrix ID (Slide) and position-related (Array) batch effects were identified by PCA as well as regression analyses of the beta values and the first three principal components. The data were analyzed by PCA to assess the variance. Principal component 1 (PC1) was associated with cell type and explained the majority of the variance (60%) in the data (p < 2 e-16; see S2 Fig). PC2 explained only 3% of the variance in the data and was associated with slide (p = 1.7 e-3). Samples with missing data in key covariates were removed and ComBat, an empirical Bayes batch-correction method as implemented in the Bioconductor sva package [47], was used to adjust for slide batch effects using a model matrix which controlled for sex, age, and ethnicity/race. Cell type proportion effects were accounted for using the proportion of leucocytes as a covariate in the analyses.

#### Calculating DNA methylation age.

The PhenoAge clock was developed from the analysis of DNAm to predict aging outcomes such as all-cause mortality, physical functioning and healthspan [48]. While PhenoAge was developed using blood samples, it has been shown to be strongly associated with chronological age in other sample types such as saliva (r = 0.81) [48]. The PhenoAge of samples in this study was estimated using the methyAge function from the ENmix R package version 1.36.08 [49].

### Data analysis

All statistical analyses were performed in RStudio. Figures were made using the ggplot2 package version 3.4.1. Pearson correlations were carried out using the cor.test function. Descriptive statistics were reported as means ± SD, unless otherwise specified, with p-values less than 0.05 reported as statistically significant. Ethnicity and race were combined into a single variable based on precedence [50] which resulted in the following ethnicity/race groups: Non-Hispanic Asian/Pacific Islander, Non-Hispanic Black/African American, Hispanic White, Non-Hispanic Other/Mixed, and Non-Hispanic White. Significance for linear regression models of DNAm data was evaluated at a false discovery rate (FDR) adjusted p-value < 0.05 using the Benjamini-Hochberg method. Version GRCh37/hg19 from the EPIC annotation manifest was used to annotate significant sites.

To test the association between EMA stress and DNAm, 5 samples with missing data in key covariates were removed and 64 samples remained after filtering. lmFit and eBayes were used to model DNA methylation as a function of average EMA stress reported during the 9-week study period prior to DNA collection, controlling for age, sex, ethnicity/race, array and cell proportions. To test the relationship between EMA stress and PhenoAge, data was restricted to the three largest ethnicity/race groups (n = 61; 43 female, 18 male) and fitted using a linear regression model, while controlling for ethnicity/race and sex. To test the association between chronic biological stress (HCC) and DNAm, 13 samples with missing data were removed prior to batch correction, leaving 56 samples for data analysis. lmFit and eBayes were implemented to model DNA methylation as a function of HCC. The analysis controlled for age, sex, ethnicity/race, array and cell proportions. To test the relationship between HCC and PhenoAge (n = 56; 38 female, 18 male), data was fitted using a linear regression model using the lm function., while controlling for age, ethnicity/race and sex.

For the DNAm analysis, in addition to genome-wide analyses, the following a priori genes related to stress were of particular interest, each with multiple CpGs represented within the Illumina EPIC array: *NR3C1* (nuclear receptor subfamily 3 group c member 1, encoding for the glucocorticoid receptor; 78 CpGs), *FKBP5* (FK506-binding protein 5; 47 CpGs), *HSP90* (heat shock protein 90; 36 CpGs), *BDNF* (brain-derived neurotrophic factor; 85 CpGs), *SLC6A4* (solute carrier family 6 member 4, encoding the serotonin transporter; 31 CpGs), *OXTR* (oxytocin receptor; 22 CpGs), *DNMT1* (DNA methyltransferase 1; 48 CpGs), and *DNMT3A* (DNA methyltransferase 3A; 111 CpGs). In addition, a sex interaction and main effect of sex in the relationship between chronic psychological and biological stress and DNA methylation was tested a priori. G*Power estimates indicated a sample size of 50 participants would be sufficient to achieve a power 0.80 with an alpha of 0.05 with a small effect size (0.20). Using a power estimation approach specifically designed for the EPIC array [51], we also determined that with 56 subjects, and a small effect size (0.20), we had an estimated power of >0.80 to detect significant differences in DNAm at 6.36% of CpG sites.

The R package missMethyl was used for the analysis of Kyoto Encyclopedia of Genes and Genomes (KEGG) defined pathways (https://www.genome.jp/kegg/pathway.html), which employs a hypergeometric test that takes into account the number of probes per gene to map genes to molecular functions [52]

This study was not pre-registered. IDAT data files, gene lists, and all variables/covariates used in the analyses are available at the following link:

https://github.com/fchampagneUT/ChronicStressDNAm.

## Results

### Summary of participants

A total of 69 participants completed the 9-week study protocol spanning January 2020 to March 2020 (n = 20 male, n = 49 female). Male participants had an average age of 23.6 (SD = 2.21) and female participants had an average age of 23.9 (SD = 3.71). The race/ethnicity groups represented in this participant sample are: Hispanic White (22.9%), Non-Hispanic White (31.4%), Asian/Pacific Islander (40%) and Other/Mixed/Black/African American (5.7%).

### Psychological stress

**Fig 1:**
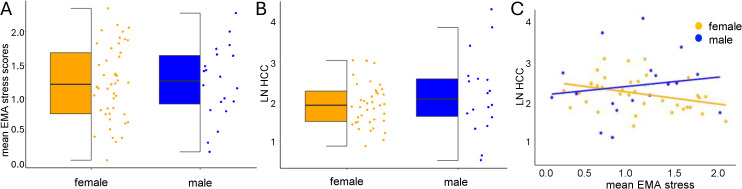
Psychological and biological stress indices in male and female participants. No sex differences were observed in (A) self-report stress (EMA stress) or in (B) natural log-transformed (LN) hair cortisol concentration (HCC). (C) EMA stress and natural log-transformed HCC were not significantly correlated.

The mean of EMA-measured stress was 1.22 (SD = .55), with a range of 0.069–2.35. The median was 1.21, indicating low to moderate self-reported psychological stress. There was no significant difference in mean EMA stress (p > 0.05) when comparing males and females (see Fig 1A). Average EMA stress and average PSS scores (mean = 15.93; SD = 6.03) were found to be significantly positively correlated (r = 0.57; p < .0001).

### Biological stress: Hair cortisol concentration (HCC)

HCC levels ranged from 1.65 to 77.5 pg/mg, the sample mean was 10.3 ± 11.88 pg/mg, and the median was 6.77 pg/mg. Natural log-transformed HCC did not differ by sex (see Fig 1B). Natural log-transformed HCC was not significantly associated with the use of hair dye, hair washing frequency, or time spent outside.

### Association between psychological and biological indicators of stress

The relationship between EMA stress (average EMA stress during the study period) and natural log-transformed HCC (assessed at the end of the study period) was not significant. However, this may be partially accounted for by a trend towards a positive association between EMA stress and natural log-transformed HCC in male participants and a trend towards a negative association between EMA stress and natural log-transformed HCC in female participants (see Fig 1C).

### Association between psychological stress and DNAm

**Fig 2:**
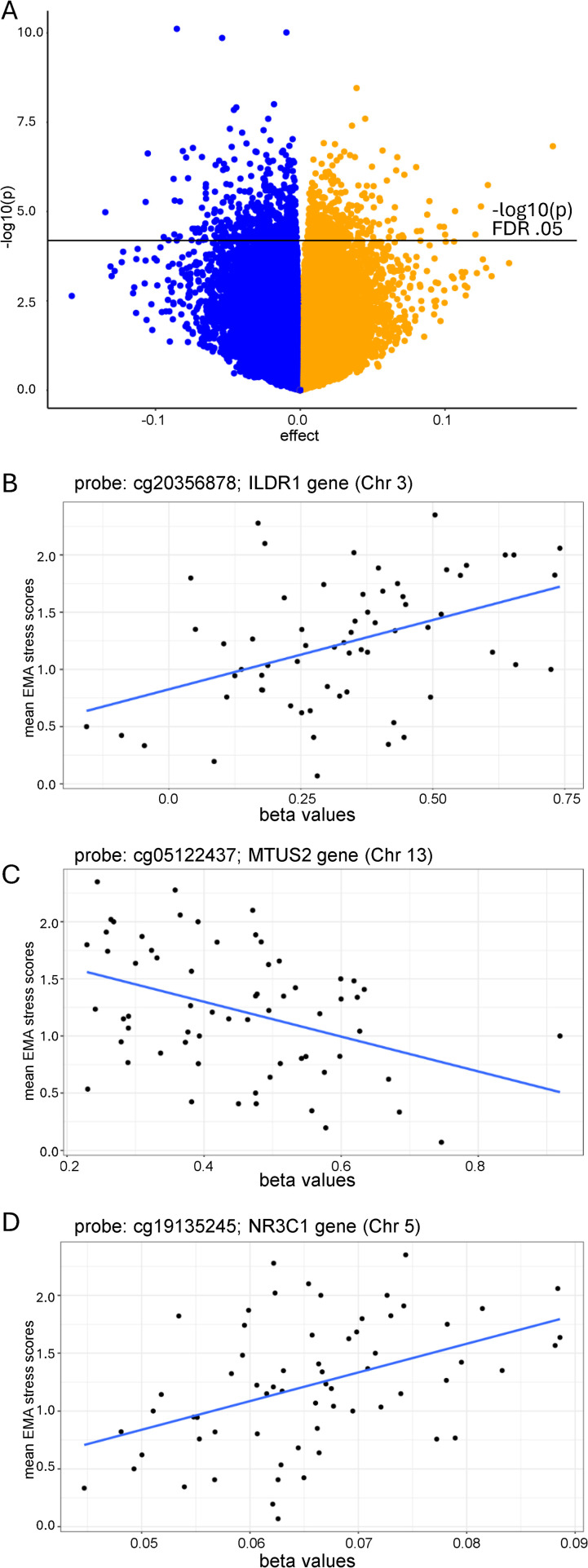
Relationship between psychological stress and DNAm. (A) Volcano plot of significant CpGs (linear mixed model testing) in the association between EMA stress and DNAm. X axis (effect) represents log2FC. The black horizontal line represents the –log10(p) value corresponding to highest raw p-value that remains significant at FDR (BH-adjusted) 0.05. Points above the line are significant. Positively associated CpGs (increased EMA stress associated with increased DNAm) are in orange. Negatively associated CpGs (increased EMA stress associated with decreased DNAm) are in blue. (B) CpG site with largest positive DNAm association with EMA stress (cg20356878). (C) CpG site with largest negative DNAm association with EMA stress (cg05122437). (D) Positive association between EMA stress and DNAm at a CpG within the *NR3C1* gene. P-values are FDR corrected to account for the multiple CpG sites included in these analyses.

When the association between self-reported psychological stress (average EMA stress during the study period) and genome-wide DNAm assessed at the end of the study period was examined, 956 significant CpG sites survived correction for multiple hypothesis testing (FDR adjusted p < 0.05) and were robust to adjustment for cell type proportions. Average DNA methylation (AvgMeth) ranged from 0.023 to 0.972, with DNAm at 564 CpGs showing a negative association and DNAm at 392 CpGs showing a positive association with psychological stress (see Fig 2A). Of the total significant CpG sites, 576 had gene annotation information indicating association with a specific gene. All significant CpG sites are presented in S1 Table. There were no EMA stress interactions with sex in the association with DNAm.

### DNAm at novel CpG sites associated with psychological stress

Overall, the effect sizes for the relationship between psychological stress and DNAm were small with cg20356878, located in a shelf region on chromosome 3 annotated to the *ILDR1* gene, showing the largest positive association with EMA stress (log2FC = 0.174, FDR adjusted p = 0.005; Fig 2B). This corresponds to an estimated ~3–4% increase in β-value DNAm per unit increase in EMA stress. With EMA stress scores ranging from 0.069 to 2.35, this equates to a total DNAm difference of approximately 6.8–9.1% across the observed range. In contrast, cg06101725 (located in an island on chromosome 13 annotated to the COG6 gene, not shown) showed the smallest effect in the relationship with EMA stress (log2FC = –0.00238), corresponding to only ~0.05% lower DNAm per stress unit, and approximately ~0.1% lower β-value DNAm across the same range, indicating a negligible effect at this locus. cg05122437 located in a shore region on chromosome 13, which was annotated to the *MTUS2* gene, exhibited the largest negative association with EMA stress (log2FC = −0.135, FDR adjusted p = 0.027; Fig 2C).

### DNAm at a priori stress-relevant genes associated with psychological stress

There were no significant CpG sites associated with the *SLC6A4*, *OXTR*, *HSP90*, *FKBP5*, *BDNF*, *DNMT1*, or *DNMT3A* genes that survived FDR correction (FDR adjusted p > 0.05). However, within the *NR3C1* gene, there was one significant CpG site that was positively associated with EMA stress (cg19135245, log2FC = 0.009, FDR adjusted p = 0.049; Fig 2D).

### Association between chronic biological stress (HCC) and DNAm

**Fig 3:**
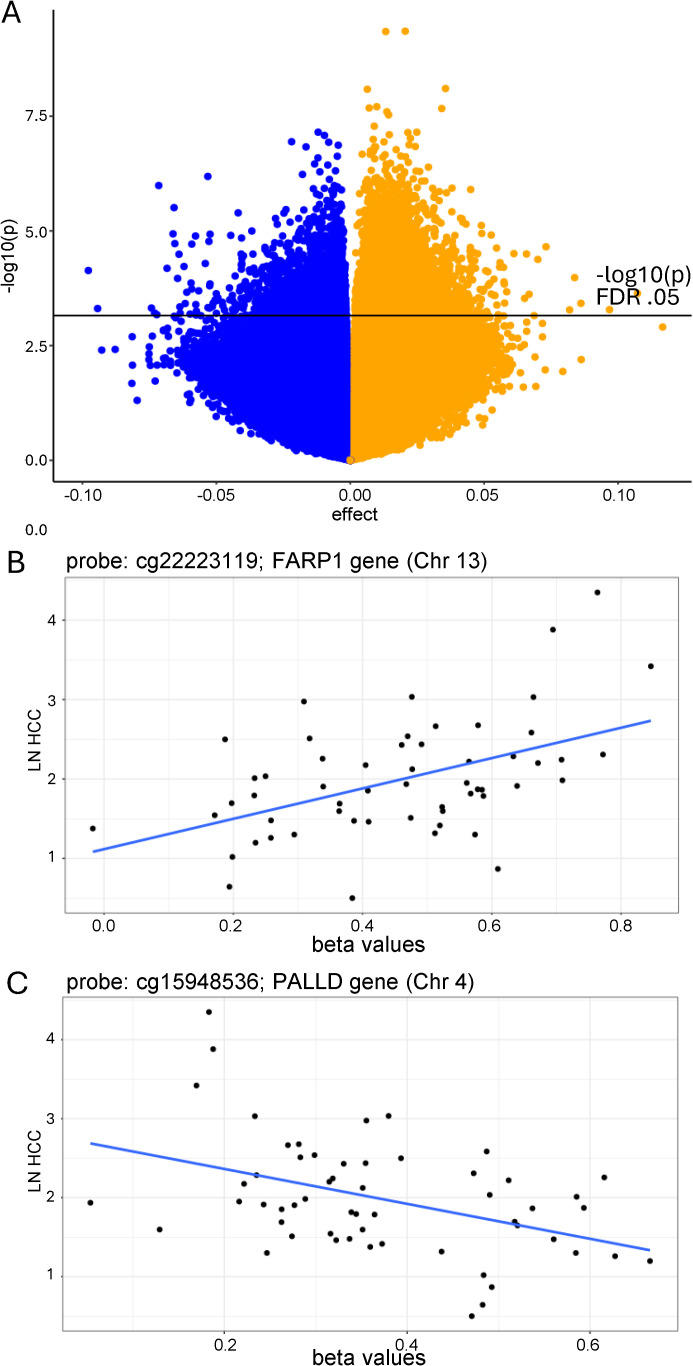
Relationship between biological stress and DNAm. (A) Volcano plot of significant CpGs (linear mixed model testing) in the association between natural log-transformed HCC and DNAm. X axis (effect) represents log2FC. The black horizontal line represents the –log10(p) value corresponding to highest raw p-value that remains significant at FDR (BH-adjusted) 0.05. Points above the line are significant. Positively associated CpGs (increased natural log-transformed HCC associated with increased DNAm) are in orange. Negatively associated CpGs (increased natural log-transformed HCC associated with decreased DNAm) are in blue. (B) CpG site with largest positive DNAm association with natural log-transformed HCC (cg22223119). (C) CpG site with largest negative DNAm association with EMA stress (cg15948536).

When the association between DNAm and natural log-transformed HCC was examined, we identified 10,335 significant CpG sites that survived FDR correction (FDR adjusted p < 0.05) and were robust to adjustment for cell type proportions. AvgMeth ranged from 0.020 to 0.983, with 2,388 CpGs showing a negative association between DNAm and natural log-transformed HCC and 7,947 CpGs showing a positive association between DNAm and natural log-transformed HCC (see Fig 3A). Among the total significant CpG sites, 7,259 had gene annotation information. All significant CpG sites are presented in S2 Table. There were no interactions between sex and natural log-transformed HCC in the association with DNAm.

### DNAm at novel CpG sites associated with chronic biological stress

Overall, the effect sizes for the relationship between biological stress and DNAm were small, with cg22223119, annotated to the *FARP1* gene (open sea on chromosome 13), showing the largest positive association between DNAm and natural log-transformed HCC (log2FC = 0.107, FDR adjusted p = 0.0356; Fig 3B). This reflects approximately ~20% higher DNAm per 10 pg/mg HCC increase. In contrast, for cg12219027 (located in an island on chromosome 19 with no gene annotation, not shown), DNAm showed a very small decrease (log2FC = –0.0014) across the biological stress range of 1.65–77.5 pg/mg HCC, equivalent to approximately 0.3% lower β-value DNAm per 10 pg/mg of stress. cg15948536, annotated to the *PALLD* gene (located in the open sea on chromosome 4), exhibited the largest negative association between DNAm and natural log-transformed HCC (log2FC = −0.098, FDR adjusted p = 0.025; Fig 3C).

### DNAm at a priori stress-relevant genes associated with chronic biological stress

**Fig 4:**
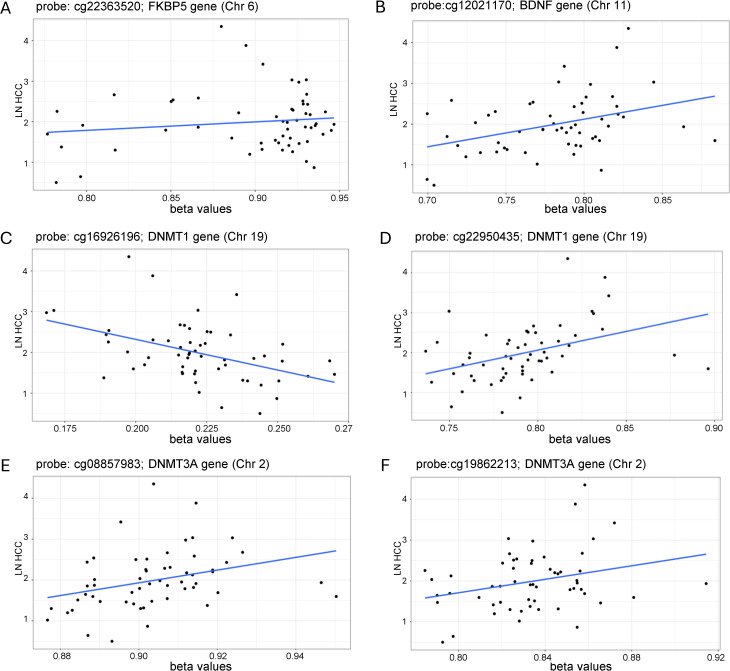
Relationship between biological stress and DNAm within stress-associated genes. (A) DNAm at cg22363520 within the *FKBP5* gene showed a significant positive association with natural log-transformed HCC. (B) DNAm at cg12021170 within the *BDNF* gene showed a significant positive relationship with natural log-transformed HCC. DNAm at two sites within the *DNMT1* gene were significantly associated with natural log-transformed HCC with (C) cg16926196 showing a negative association and (D) cg22950435 showing a positive association. DNAm at two sites within the *DNMT3*A gene were significantly positively associated with natural log-transformed HCC: (D) cg08857983 and (E) cg19862213. P-values are FDR corrected to account for the multiple CpG sites included in these analyses.

There was no significant CpG site DNAm associated with natural log-transformed HCC within the *NR3C1*, *HSP90*, *SLC6A4*, and *OXTR* genes (p > 0.05). However, we found a relationship between natural log-transformed HCC and DNAm at several CpG sites related to other a priori stress-relevant genes which survived FDR correction (FDR adjusted p < 0.05) and were robust to adjustment for cell type proportions. The *FKBP5* gene (open sea region on chromosome 6) had a CpG site positively associated with natural log-transformed HCC (cg22363520, log2FC = 0.019, FDR adjusted p = 0.014; Fig 4A). The *BDNF* gene (open sea of chromosome 11) had a CpG site positively associated with natural log-transformed HCC (cg12021170: log2FC = 0.026, FDR adjusted p = 0.030; Fig 4B). Within the *DNMT1* gene (chromosome 19), there were two CpG sites associated with natural log-transformed HCC, one with a negative association (cg16926196: log2FC = −0.014, FDR adjusted p = 0.023; Fig 4C) and one with a positive association (cg22950435: log2FC = 0.019, FDR adjusted p = 0.016; Fig 4D). Within the *DNMT3A* gene (chromosome 2), two CpG sites were positively associated with natural log-transformed HCC (cg08857983: log2FC = 0.008, FDR adjusted p = 0.048, Fig 4E; cg19862213: log2FC = 0.011, FDR adjusted p = 0.048, Fig 4F).

### Convergence in differential CpG DNAm in association with psychological and biological stress

Among the CpG sites that had a significant association between DNAm and natural log-transformed HCC, 16 survived FDR correction (FDR adjusted p < 0.05) and were robust to adjustment for cell type proportions when examining the association between EMA stress and DNAm (see Table 1). Interestingly, despite overlap in the CpG sites exhibiting an association with both natural log-transformed HCC and EMA stress, the direction of association was in the opposite direction in these comparisons, such that sites observed to be hypomethylated in association with EMA stress where found to be hypermethylated in association with natural log-transformed HCC and sites observed to be hypermethylated in association with EMA stress where found to be hypomethylated in association with natural log-transformed HCC.

**Table 1:** CpGs with differential DNAm associated with both EMA and natural log-transformed HCC.

UCSC RefGene Name	CpG	Chr	Pos	Log2FC (EMA - DNAm)	p value (EMA-DNAm)	FDR p (EMA-DNAm)	Log2FC (HCC - DNAm)	p value (HCC-DNAm)	FDR p (HCC-DNAm)
PTGER4	cg01091117	5	40682333	0.028	4.21e-5	0.043	−0.023	1.20e-4	0.029
HLA-C	cg05030953	6	31241000	0.123	1.81e-6	0.013	−0.072	6.69e-4	0.049
N/A	cg16745057	7	51673444	0.010	1.91e-6	0.013	−0.007	4.06e-4	0.042
PMEPA1	cg26912636	20	56286391	0.025	1.54e-5	0.031	−0.016	5.65e-4	0.047
ENTPD2	cg02167771	9	139944139	−0.041	1.20e-6	0.010	0.031	8.84e-6	0.014
N/A	cg03623401	16	12043656	−0.032	3.64e-5	0.041	0.031	1.18e-6	0.008
N/A	cg11374582	17	61927086	−0.027	1.54e-5	0.031	0.021	6.21e-5	0.024
N/A	cg12033288	10	102381293	−0.020	9.20e-6	0.026	0.014	7.14e-5	0.025
SLC30A9	cg13237657	4	41992219	−0.006	6.02e-5	0.049	0.005	1.59e-4	0.032
N/A	cg13344095	6	2623335	−0.079	3.07e-7	0.006	0.052	1.53e-4	0.031
N/A	cg18291422	6	147171560	−0.011	2.59e-5	0.036	0.009	8.65e-6	0.014
ELMO3	cg22417309	16	67233653	−0.022	1.58e-5	0.031	0.016	9.71e-5	0.027
GSTM4	cg07569194	1	110200960	−0.012	1.99e-5	0.033	0.008	4.43e-4	0.043
HPCAL1	cg11855694	2	10499528	−0.014	4.97e-5	0.046	0.011	6.39e-6	0.013
C11orf49	cg24347903	11	46958091	−0.022	5.46e-5	0.047	0.018	1.18e-4	0.029
N/A	cg25756867	3	180042726	−0.018	5.06e-5	0.046	0.011	3.26e-4	0.039

### Biological pathways implicated in analyses of the association between psychological stress, biological stress and DNAm

KEGG was implemented to identify biological pathways based on the genes identified in the DNAm analyses. Input for the pathway analysis included CpGs with FDR adjusted p-values < 0.05 from the association between DNAm and EMA (N = 956) and between DNAm and natural log-transformed HCC (N = 10,335). Top KEGG pathways for the DNAm-EMA association included metabolism (FDR p = 5.50 e-9), cancer (FDR p = 1.78 e-4), MAPK signaling (FDR p = 1.94 e-4), neurodegeneration (FDR p = 1.94 e-4), and Rap1 signaling (FDR p = 4.43 e-4; S3 Table). Top KEGG pathways for the DNAm-natural log-transformed HCC association included metabolism (FDR p = 5.67 e-10), focal adhesion (FDR p = 2.28 e-6), MAPK signaling (FDR p = 2.31 e-5), neurodegeneration (FDR p = 1.62 e-4), Ras signaling (FDR p = 1.62 e-4) and Rap1 signaling (FDR p = 1.83 e-4; S4 Table).

### Association between psychological stress, biological stress and epigenetic aging

**Fig 5:**
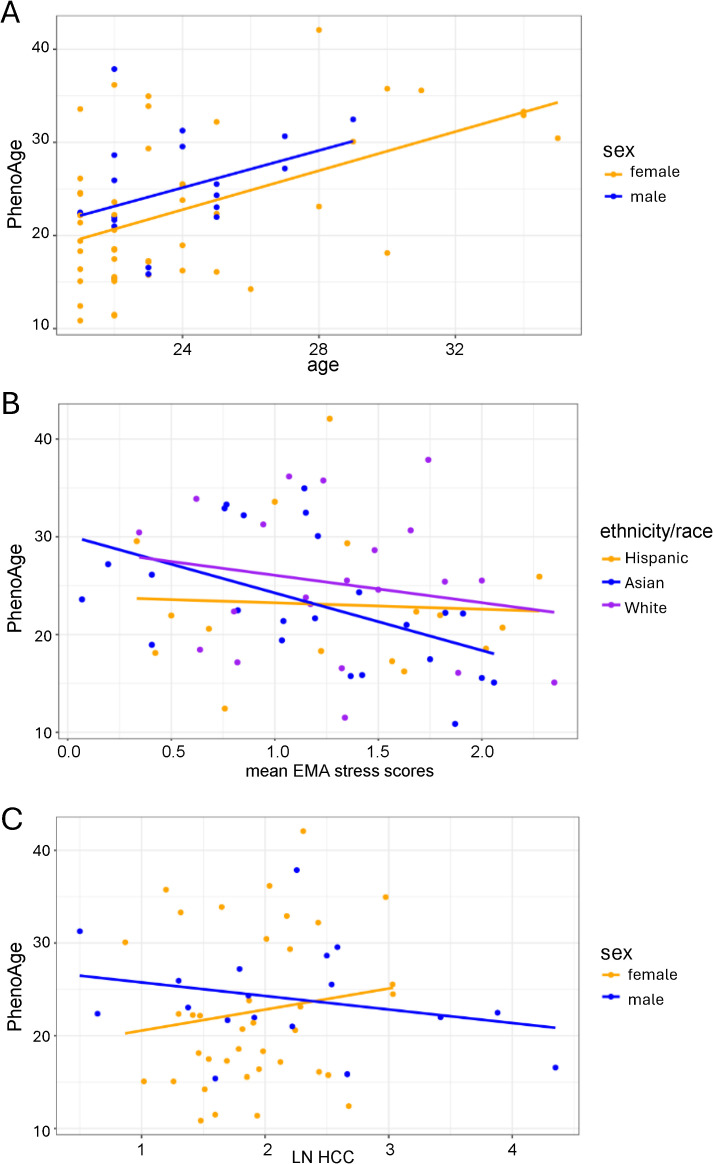
Epigenetic age and its association with psychological and biological stress. (A) PhenoAge had a significant positive association with chonological age (r = 0.47). (B) A negative relationship between EMA stress and PhenoAge was found when controlling for ethnicity/race and sex. (C) A negative relationship between natural log-transformed HCC and PhenoAge was found in males.

PhenoAge (mean: 23.36 ± 7.33, median: 22.34) was moderately associated with chronological age (r = 0.47; Fig 5A). When the association between EMA stress and PhenoAge was examined, EMA stress was negatively associated with PhenoAge (log2FC = −3.414, SE = 1.630, t = −2.094, p = 0.041), controlling for ethnicity/race and sex (Fig 5B). When the association between natural log-transformed HCC and PhenoAge was examined, a significant interaction between sex and natural log-transformed HCC emerged when the model controlled for age and ethnicity/race (log2FC = −5.216, SE = 2.572, t = −2.028, p = 0.048) with males showing a consistently negative relationship between natural log-transformed HCC and PhenoAge (Fig 5C).

## Discussion

The association between stress and the epigenome has been hypothesized to be a critical pathway linking exposure to stress and adversity to long-term health outcomes. However, disentangling the broad definitions of stress that have been used to examine these relationships is critical to understanding the mechanisms by which stress-induced effects confer health risks. In the current study, we examined the association between psychological stress vs. biological stress and variation in the human epigenome in a sample of healthy young adults participating in a longitudinal study. Consistent with prior studies [34], we found that the perception of stress (psychological stress) and HCC (biological stress) were not significantly correlated, though sex-specific effects may be impacting this relationship. However, both perception of stress and HCC were associated with genome-wide differential DNAm, with generally non-overlapping associations with CpG DNAm and unique general trends in differential DNAm (psychological stress associated with hypomethylation; biological stress associated with hypermethylation). The unique epigenomic associations with psychological vs. biological stress were further demonstrated by the opposing direction of associations (i.e., hypomethylation vs. hypermethylation) among the 16 CpG sites that are significantly associated with both forms of stress. Analyses of DNAm at CpG sites within stress-related genes similarly indicated that psychological and biological stress have unique associations with the epigenome, though both indices of stress were generally associated with increased DNAm of stress-related genes. Analyses of potential biological pathways implicated in the DNAm-stress relationship identified overlapping pathways (i.e., metabolism, MAPK signaling, neurodegeneration, Rap1 signaling), though given the opposing directions of the associations with DNAm, up-regulation vs. down-regulation corresponding to psychological vs. biological stress may emerge (assuming functional down-regulation with hypermethylation). Finally, analyses of epigenetic aging, a measure that utilizes genome DNAm to predict age and age-related health outcomes [48], indicated that both psychological and biological stress were associated with a decrease in epigenetic age, though in the case of biological stress, this relationship was specific to males. These analyses account for confounding variables by adjusting for sex, age, ethnicity/race, estimated cell proportions and batch effects. In addition, stringent probe filtering criteria were applied to the data to reduce the number of hypothesis tests and false positives with reported effects being robust to adjustments for multiple hypothesis testing. Overall, these findings suggest that psychological stress and biological stress have unique associations with genome-wide DNAm and highlight the complex relationship between stress and the epigenome.

### Complex relationship between psychological and biological stress

While it is generally assumed that elevated perception of stress and elevated HPA activity co-occur, individual differences in the impact of stress are well established. Our previous studies indicate that during exposure to an acute psychological stressor, though both self-report stress and salivary cortisol increase, these measures of stress are not correlated within individuals [53]. In the current study of chronic stress, we found that self-report stress and HCC are not significantly correlated. However, stratifying the analyses by sex indicates that there may be sex-specific trends, with males showing a trend for a positive relationship and females showing a trend for a negative relationship. A lack of significant correlation between HCC and psychological stress has been previously been observed [34]. While there is support for an association between exposure to chronic stressors and HCC [30], it would appear that the perception of those stressors represents a unique socioemotional-cognitive variable that diverges from HPA measures. A similar disassociation has been observed in analyses of the effects of interventions that reduce perception of stress during exposure to an acute stressor [54] and correspondence between psychological and biological stress during an acute stress exposure appears to be the exception rather than the norm [55]. Methodological variables may account for inconsistencies within the literature, though non-linear relationships between self-report perception of stress and HCC have also been observed. An inverted U shaped function may better describe this relationship wherein HCC increases in association with elevated perceived stress but then decreases at very high levels of perceived stress [56]. These non-linear relationships in combination with positive and negative feedback loops within the HPA axis may account for the complex relationship between cortisol levels and mental health outcomes [30].

Sex differences in both psychological and biological stress have been previously documented, though individual differences in stress responses create significant variability within this literature. Across the lifespan, females have been reported to have higher perceived stress when compared to males [57,58]; a phenomenon that has been hypothesized to contribute to the development of mood disorders [58,59]. In contrast, cortisol levels have been found to be elevated in males compared to females under conditions of acute stress [60] and HCC has been found to be increased in males compared to females [27]. We did not observe sex differences in psychological stress or HCC in the participants of this study. However, overall levels of HCC were found to be similar to those reported in healthy young adults and consistent with previous studies, male participants had a wider range of HCC values [27,33]. Sex-specific relationships between psychological and biological stress are suggested within our analyses, with males exhibiting a positive relationship between self-report stress and HCC, though larger sample sizes would be necessary to determine whether these effects are robust. Sex-specific effects are likely an important consideration within studies of stress and may be particularly relevant within studies of DNAm. We did not observe interactions between sex and stress in the association with genome-wide DNAm, but sex-specific effects were observed in the analysis of PhenoAge, indicating that elevated HCC was associated with reduced PhenoAge in males but not in females. The mechanism driving these sex specific effects is not known, however hormonal, genetic, and environmental factors likely collectively influence the way males compared to females respond to stress.

### Genome-wide analyses of the association between DNAm and stress

Among the > 900 CpG sites that we identified as having differential DNAm in association with psychological stress, a CpG site within the immunoglobulin like domain containing receptor 1(*ILDR1*) gene had the strongest positive association. Differential DNAm in this gene has not previously been found associated with stress. Studies of altered DNAm in this gene have implicated its role in Meniere disease (MD) [61] and multiple sclerosis [62], with mutations in this gene associated with hearing loss [63]. A CpG site within the microtubule associated scaffold protein 2 (*MTUS2*) gene had the strongest negative association with psychological stress. Differential DNAm in the *MTUS2* gene has previously been found in blood samples from Parkinson’s disease patients compared to healthy controls [64] and genomic studies implicate *MTUS2* late-onset Alzheimer’s disease [65]. Studies of acute and chronic stress in mice suggest that differential DNAm within the *Mtus2* gene may serve as an “epigenetic primer” to stress exposure and its neurobiological and behavioral consequences [66]. While these genes and CpG sites are novel within the context of psychological stress, it is unlikely that any specific gene pathway would account for significant variance in psychological stress. However, the genes and CpG sites identified (see S1 Table) may be a useful foundation for future exploration, particularly within research designs in which causal inference can be established.

Examination of the association between HCC and DNAm revealed >10,000 significant CpG sites with less than a 0.002% overlap with CpG sites indicated in analyses of psychological stress (see Table 1). A CpG site within the *FARP1* gene (FERM, ARH/RhoGEF and pleckstrin domain protein 1) exhibited the strongest positive association with HCC. FARP1 has been shown to serve a role in neurodevelopment, with specific effects on dendritic growth and complexity [67,68]. Interestingly, chronic glucocorticoid exposure has been found to decrease dendritic growth and complexity during development and in adulthood [69,70], suggesting a possible role for *FARP1* in stress-associated neural functioning. DNAm in the *PALLD* gene (palladin, cytoskeletal associated protein) had the most significant negative association with HCC. *PALLD* encodes the palladin protein which is involved in cytoskeleton dynamics and cellular morphagenesis [71]. Upregulation of *PALLD* expression has been found associated in obesity [72] and the palladin protein has been implicated in mechanistic pathways conferring risk for cancer [73] and cardiovascular disease [74]. Similar to the analysis of psychological stress, it is unlikely that CpG DNAm at any given gene/loci would account for significant variation in biological stress. The extensive list of genes/loci revealed from these analyses (see S2 Table) could be the focus of future research examining the potential mechanisms that account for altered HPA response to chronic stress. The KEGG pathway analyses suggest that genes involved in metabolism may be particularly sensitive to both psychological and biological stress, which aligns with the established association between chronic stress and metabolic dysregulation [75,76]. However, other pathways identified, such as MAPK signaling and neurodegeneration, have also been associated with stress [77,78] and may be important contributors to health outcomes.

We found both hypermethylation and hypomethylation in association with psychological and biological stress. However, among the significantly differentially methylated CpGs, psychological stress was associated with a greater likelihood of hypomethylation and HCC was associated with a greater likelihood of hypermethylation. These general trends in DNAm suggest that psychological stress and biological stress operate via unique mechanisms that involve global epigenetic processes. These global processes likely involve DNA methyltransferase (DNMT) or ten-eleven translocation (TET) enzyme activity that can promote DNAm or demethylation respectively [79,80]. In rodents, chronic stress exposure has been associated with decreased *Dnmt* expression (*Dnmt3a*) and a shift toward an increased DNAm at CpGs in association with stress [81]. TET enzymes may modulate susceptibility to chronic stress and hydroxymethylation has been found to decrease globally within the prefrontal cortex in response to chronic stress [82]. These findings highlight the dynamic shifts in DNAm that can occur in response to stress and indicate that prediction of the direction of the global shift in DNAm will be challenging due to the potential opposing effects of DNMTs vs. TET enzymatic processes. For example, while we observe hypermethylation associated with HCC, previous studies in humans suggest that hypercortisolism is associated with a global reduction in DNAm [83].

### Chronic stress and DNAm within stress-related genes

Candidate gene studies have served as the foundation for the exploration of the relationship between stress and DNAm, with a particular emphasis on genes involved in HPA activation. Our analyses indicate a significant association between stress and DNAm within these candidate genes, with distinct associations in psychological vs. biological stress. We found that chronic psychological stress was associated with increased DNAm within the *NR3C1* gene at a CpG site associated with the gene promotor region of this gene. Increased DNAm of the *NR3C1* gene promotor has been observed in response to low levels of maternal care [84,85], chronic stress during infancy [86], and in association with prenatal and postnatal adversity in humans [87–89]. The particular CpG site we identified as having a positive association with psychological stress was previously found to have reduced DNAm in children in response to cognitive behavioral therapy of mothers [90]. Epigenetic plasticity in the *NR3C1* gene is well-established and DNAm within this gene may shift in response to characteristics of the social environment. However, it is important to note that while studies in animals do suggest that chronic stress can induce alteration in DNAm in the *NR3C1* gene, variation in DNAm within this gene may also lead to changes in HPA functioning. Expression of hippocampal *NR3C1* is critical for negative feedback within the HPA axis, and reduced *NR3C1* expression via increased DNAm may lead to more prolonged physiological responses to stressors [91,92]. Our research design is not able to establish the causal direction of the association between psychological stress and DNAm, though it is likely that there is bidirectional interplay throughout the lifespan with contributes to this association.

In the current study, chronic biological stress was found to be significantly associated with DNAm within the *FKBP5*, *BDNF*, *DNMT1*, and *DNMT3A* genes. *FKBP5* encodes a co-chaperone of HSP90 and contributes to the translocation of the glucocorticoid receptor to the cell nucleus and subsequent binding to glucocorticoid response elements [93,94]. Previous studies in mice indicate that chronic glucocorticoid exposure induces increased *Fkbp5* expression across multiple tissues and an overall decrease in *Fkbp5* DNAm [95]. Similar findings have been observed in humans that examine the association between cortisol levels and *FKBP5* expression and DNAm [96]. The specific CpG site that we have identified as being positively associated with HCC has previously been shown to be hypomethylated amongst children that experience adverse physical and social environments as neonates [97]. Conversely, studies in healthy young adults have not demonstrated a significant relationship between *FKBP5* DNAm and cortisol levels, measured either during acute stress exposure or via HCC [98]. Some critical issues to address in interpretation of this body of work is establishing the relationship between differential *FKBP5* DNAm and gene expression, the tissue specificity of stress-associated effects on *FKBP5* DNAm, sex-specificity of effects, and the role of genetic variations within the *FKBP5* in predicting associations between *FKBP5* DNAm and stress. Similar issues are relevant to interpretation of the positive association between HCC and DNAm in the *BDNF* gene. Though this finding is consistent with previous reports [32], and with evidence that chronic stress exposure results in increased DNAm within the *BDNF* gene [87,99,100], genetic variants within the *BDNF* gene may moderate the effects of chronic stress [101]. DNAm within *BDNF* is also highly dynamic, creating challenges to establishing consistent findings across studies. However, hypomethylation of the CpG site within the *BDNF* gene identified in the current study has previously been observed in response to an exercise intervention that decreased physiological markers of stress [102]; a finding consistent with the interpretation that stress has a positive association with *BDNF* DNAm at this site.

The role of DNMTs in stress-associated effects has been previously demonstrated. In mice, chronic stress has been found to increase the expression of *DNMT1* and *Dnmt3a* in the nucleus accumbens [103,104], whereas an increase in *DNMT1* expression has been observed in vitro and within the mouse hippocampus following treatment with glucocorticoids [105]. Chronic prenatal stress in rats has been demonstrated to increase placental *Dnmt3a* and cortical *DNMT1* expression [106]. The associations we observe between HCC and DNAm within these methyltransferase genes may implicate *DNMT* expression as a potential mediator of the relationship between biological stress and genome-wide DNA methylation, though the transcriptional consequences of the differential DNAm we have observed in these genes would need to be established.

### Stress and epigenetic aging

Epigenetic aging as an indicator of processes contributing to healthspan and longevity has been increasingly explored within the context of studies of chronic stress and adversity. Adverse childhood experiences are associated with epigenetic age acceleration in later life [107] and it would generally be predicted that chronic stress (psychological and biological) would exert significant biological “wear and tear” that would be reflected in measures epigenetic age acceleration and subsequently increased risk of poor physical and psychological health outcomes [108]. However, using the PhenoAge epigenetic clock [48], we found a negative association between both the perception of stress and HCC across a 9-week period and PhenoAge. Similar to our findings, a previous examination of the association between perceived stress (PSS) and epigenetic age (including PhenoAge) in older adults found a negative association, though this association became non-significant when controlling for covariates such as lifestyle and ancestry [109]. In contrast, chronic stressors such as low socioeconomic status in childhood, have been associated with PhenoAge acceleration in later life [110]. There is limited evidence of a relationship between cortisol and epigenetic age acceleration; HCC has yet to be explored in this context. We have previously observed that levels of cortisol reactivity but not psychological stress reactivity during an acute stressor are positively associated with accelerated PhenoAge in females [14] The current state of knowledge regarding the factors that promote epigenetic age acceleration vs. deceleration is limited and the lack of consistency in the emerging literature suggests that complex processes underly the relationship between stress, epigenetic aging, and health outcomes. For example, it has been reported that though cumulative life stress is predictive of epigenetic age acceleration, this effect is observed in some epigenetic clocks (i.e., GrimAge and DunedinPace) but not others (i.e., PhenoAge) and only observed in females [111]. A critical issue to consider when implementing DNAm-based aging clocks is whether they are appropriate predictors across all race/ethnicity groups, as these clocks have generally been developed using individuals of European ancestry [112]. Though we have included race/ethnicity as covariates in our analyses, this does not address underlying bias in the predictive models being utilized by the clocks.

### Limitations and future directions

Though the genome-wide approach and longitudinal design of the study coupled with EMA-based assessments of psychological stress during a time period coinciding with HCC assay are significant strengths of the study, there are limitations associated with the current study. Saliva is a non-invasive tissue that is optimal for longitudinal studies in humans. However, DNAm in the epithelial and immune cells present in these samples are unlikely to be highly concordant with the brain, which may limit any inference that the reported variation in DNAm may impact neural functioning. However, given the systemic effects of stress, we propose that many of the reported epigenetic associations occur across tissue types. In addition, though DNAm can alter gene expression, it is unknown whether the variation in DNAm reported in the current study exerts a transcriptional effect. Establishing the functional consequences of the observed variation in DNAm would be an important step towards understanding the potential phenotypic consequences and the direction of effects on predicted biological processes. There are also genetic influences on DNAm that we are unable to assess in the current study and may impact response to stress [113]. Our analyses were restricted to the PhenoAge epigenetic clock and future work should consider the associations with other epigenetic clocks that may be more appropriate in healthy young adults. Finally, since we did not manipulate stress in the current study, it is not possible to establish the causal direction of the observed associations between stress and DNAm. While it is plausible that stress (psychological and biological) has an impact on DNAm, it is equally likely that variation in DNAm can impact an individual’s response to stress. Future studies exploring the potential of these bidirectional influences may provide critical insights into the relationship between stress, the epigenome, and health outcomes.

## Supporting information

S1 Fig Expected compared to observed p values in the analyses of the association between DNAm and stress variables. Q-Q plots of p values for analyses of the association between (A) DNAm and psychological stress (EMA) and (B) DNAm and HCC.
(TIF)

S2 Fig Results of a principal component analyses (PCA) of variation in DNA methylation. PC1 accounted for the majority of variance and associated with cell type proportions. PC2 was associated with slide.
(TIF)

S1 Table All significant CpG sites in the analysis of the association between DNAm and psychological stress. Includes information regarding CpG Site, log fold change, average methylation, p value, FDR adjusted p value, chromosome, position, location type, and gene annotation.
(XLSX)

S2 Table All significant CpG sites in the analysis of the association between DNAm and HCC. Includes information regarding: CpG Site, log fold change, average methylation, p value, FDR adjusted p value, chromosome, position, location type, and gene annotation.
(XLSX)

S3 Table Top KEGG pathways identified in the association between DNAm and psychological stress. Includes information regarding pathway description, number of genes in KEGG term, number of genes differentially methylated, p value, and FDR adjusted p value.
(XLSX)

S4 Table Top KEGG pathways identified in the association between DNAm and HCC. Includes information regarding pathway description, number of genes in KEGG term, number of genes differentially methylated, p value, and FDR adjusted p value.
(XLSX)
