# Supplementary figures and images for: Association between chronic stress and the epigenome: Exploration of psychological and biological stress
Source: PLoS One. 2026 Apr 6;21(4):e0346517. doi: 10.1371/journal.pone.0346517 (PMC13052847; doi:10.1371/journal.pone.0346517)

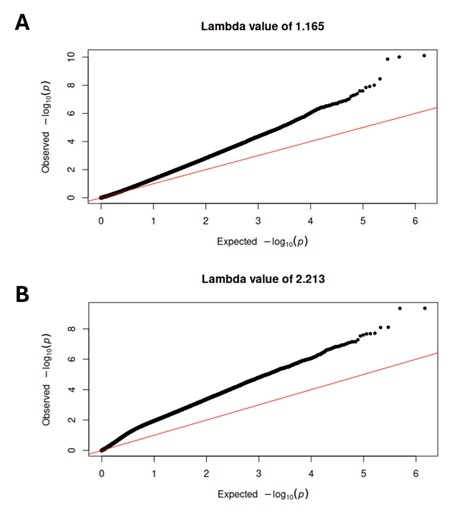

Supplement: S1 Fig — Q-Q plots of p values for analyses of the association between (A) DNAm and psychological stress (EMA) and (B) DNAm and HCC. (TIF) [file pone.0346517.s001.tif]

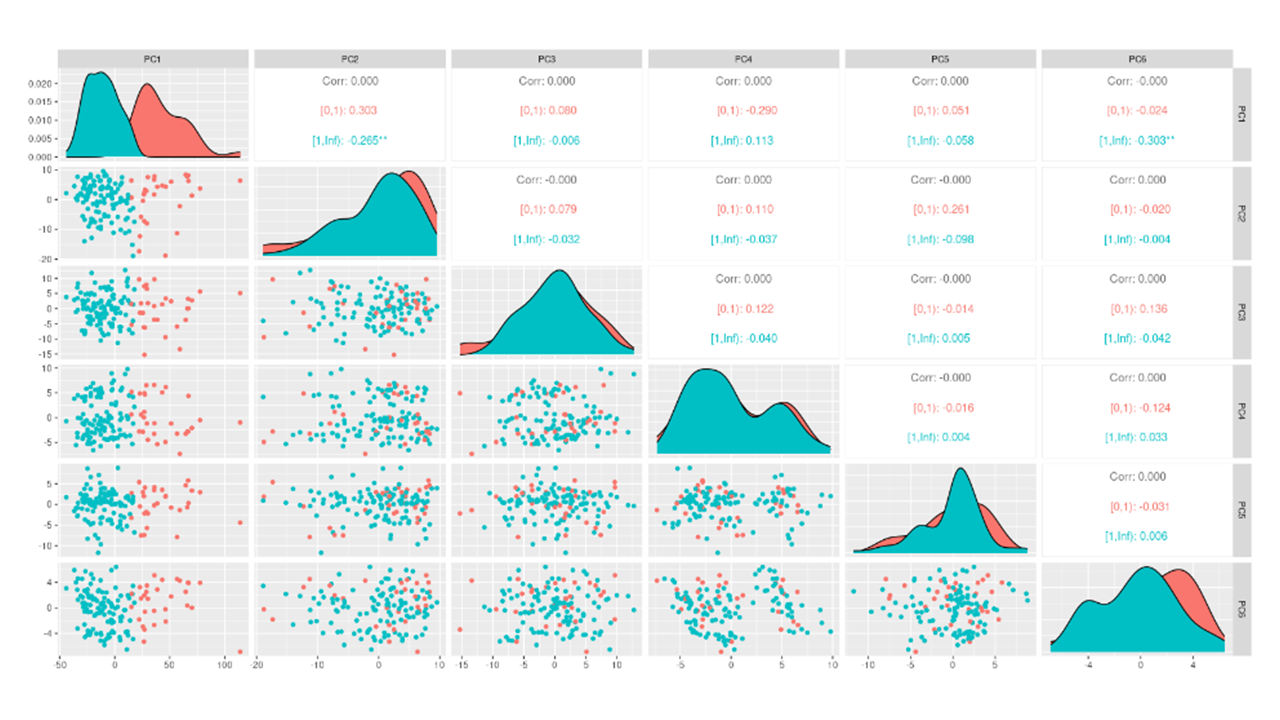

Supplement: S2 Fig — PC1 accounted for the majority of variance and associated with cell type proportions. PC2 was associated with slide. (TIF) [file pone.0346517.s002.tif]
